# The Metabolic Chemical Reporter Ac_4_6AzGal Could Incorporate Intracellular Protein Modification in the Form of UDP-6AzGlc Mediated by OGT and Enzymes in the Leloir Pathway

**DOI:** 10.3389/fchem.2021.708306

**Published:** 2021-10-12

**Authors:** Jiajia Wang, Biao Dou, Lu Zheng, Wei Cao, Peiyu Dong, Yingyi Chen, Xueke Zeng, Yinhang Wen, Wenxuan Pan, Jing Ma, Jingying Chen, Xia Li

**Affiliations:** ^1^ Joint National Laboratory for Antibody Drug Engineering, the First Affiliated Hospital of Henan University, School of Basic Medicine Science, Henan University, Kaifeng, China; ^2^ State Key Laboratory of Medicinal Chemical Biology, Haihe Education Park, Nankai University, Tianjin, China; ^3^ School of Pharmacy, Institute for Innovative Drug Design and Evaluation, Henan University, Kaifeng, China

**Keywords:** OGT/OGA, Leloir pathway, GALE, GALT, Ac46AzGalactose, metabolic chemical reporter

## Abstract

Galactose is a naturally occurring monosaccharide used to build complex glycans that has not been targeted for labeling as a metabolic reporter. Here, we characterize the cellular modification of proteins by using Ac_4_6AzGal in a dose- and time-dependent manner. It is noted that a vast majority of this labeling of Ac_4_6AzGal occurs intracellularly in a range of mammalian cells. We also provided evidence that this labeling is dependent on not only the enzymes of OGT responsible for O-GlcNAcylation but also the enzymes of GALT and GALE in the Leloir pathway. Notably, we discover that Ac_4_6AzGal is not the direct substrate of OGT, and the labeling results may attribute to UDP-6AzGlc after epimerization of UDP-6AzGal *via* GALE. Together, these discoveries support the conclusion that Ac_4_6AzGal as an analogue of galactose could metabolically label intracellular O-glycosylation modification, raising the possibility of characterization with impaired functions of the galactose metabolism in the Leloir pathway under certain conditions, such as galactosemias.

## Introduction

O-GlcNAc modification of serine and threonine residues of intracellular proteins is ubiquitous in eukaryotic cells (Schwein and Woo, 2020; Zol-Hanlon and Schumann, 2020; Ma et al., 2021). The process of O-GlcNAc in the protein cycle is controlled by two highly conserved enzymes: O-GlcNAc transferase (OGT) and O-GlcNAcase (OGA). OGT transfers UDP-GlcNAc to the target protein, and O-GlcNAcase hydrolyzes residues. O-GlcNAcylation, as an important glycosylation modification, is involved in many important cellular biological processes, including transcription, translation, and signal transduction, and plays an important role in human physiological and pathological processes. Abundant studies have shown that the homeostasis of O-GlcNAc levels is related to the occurrence and development of many diseases, such as diabetes, neurodegenerative diseases, cancer, and some autoimmune diseases. Therefore, it exhibited a great significance for the detection and identification of O-GlcNAc glycosylated proteins (Vocadlo, 2012; Chaiyawat et al., 2014; Worth et al., 2017; Hanover et al., 2018; Nagy et al., 2019; Estevez et al., 2020).

Metabolic chemical reporters (MCRs) of sugar metabolites with bio-orthogonal functional groups have become a strategy widely used to visualize and identify glycoproteins. This strategy involves synthesizing non-natural monosaccharide analogues with bio-orthogonal functional groups, such as alkyne or azide analogues (Wang et al., 2019; Wang and Mooney, 2020). These small molecules are used to treat living cells and animals, which are converted into nucleotide donor sugars by the metabolism in the body and incorporated into glycans by glycosyltransferases (Chuh et al., 2016). Finally, biotin or the fluorescent tag is labeled on the glycan-modified protein through bio-orthogonal click chemical reaction to realize visualization and enrichment of glycoproteins. Currently, an increasing number of peracetylated O-GlcNAc analogues for O-GlcNAc metabolic markers have been developed and characterized, such as modification at the 2-position of HexNAc (Vocadlo et al., 2003; Boyce et al., 2011; Tan et al., 2018) or at the 4-/6-sites (Chuh et al., 2014; Li et al., 2016; Chuh et al., 2017; Darabedian et al., 2018; Guo et al., 2019), and all the reported functionalized GlcNAc analogues demonstrated satisfactory selectivity and efficiency, which exhibited a wide range of substrate tolerance of OGT (Li et al., 2018). Meanwhile, any nonanalogues of GlcNAc, 6-azido-6-deoxy-glucose (6AzGlc) (Darabedian et al., 2018), and 2-azido-2-deoxy-glucose (2AzGlc) (Zaro et al., 2017) are also reported as the substrates of OGT to modify O-GlcNAc proteins.

**SCHEME 1:**
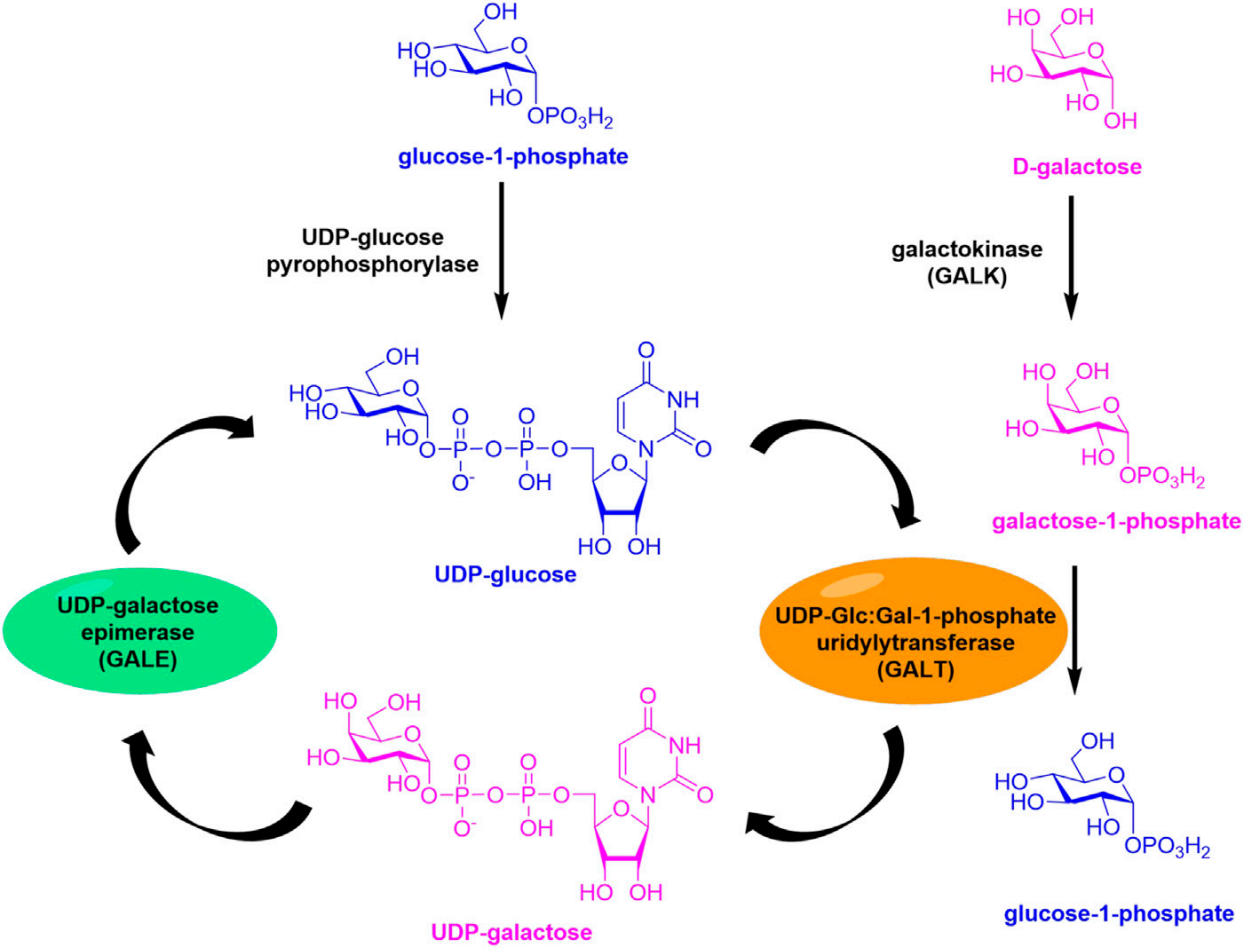
Leloir pathway of the galactose metabolism. The highly conserved Leloir pathway is composed of three enzymes: galactokinase (GALK), galactose-1-phosphate uridylyltransferase (GALT), and UDP galactose 4′-epimerase (GALE).

Galactose is a monosaccharide with the same chemical formula as glucose, differing only in the position of the hydroxyl group at the 4-site. The main route of the galactose metabolism, the Leloir pathway, results in glucose-1-phosphate for glycolysis, which is accomplished by the action of three key enzymes (Scheme 1) (Holden et al., 2004; McAuley et al., 2016). The first enzyme, galactokinase (GALK), catalyzes the phosphorylation of galactose to galactose-1-phosphate (Gal-1P); the second enzyme, galactose-1-phosphate uridylyltransferase (GALT), cleaves UDP-glucose to release glucose-1-phosphate, and the retaining UMP is received by Gal-1P to afford UDP-galactose; the third one, UDP-galactose 4′-epimerase (GALE), interconverts UDP-Galactose and UDP-Glucose. The important metabolic role of enzymes has attracted significant research attention. Defects in human enzymes lead to diseases stated referred to as galactosemia, which was caused by the incapability of catabolizing dietary Gal *via* the Leloir pathway. Moreover, defects in the breakdown of Gal-related glycans result in several lysosomal storage diseases. Metabolic oligosaccharide engineering provides a powerful tool to investigate the roles of various monosaccharides through the copper-catalyzed azide-alkyne cycloaddition (CuAAC) in combination with appropriate tags. However, few documents disclose the roles of Gal including its analogues in the glycosylation changes in development and disease progression due to the strict substrate specificity. Recently, Baskin’s group developed 6-alkynyl UDP-Gal for imaging N-glycans in developing zebrafish *in vivo* (Daughtry et al., 2020). In addition, they did not find any obviously metabolic labeling of cell-surface glycans using peracetylated 6-azide or 6-alkynyl Gal, probably resulting from the limited tolerance of GALK to unnatural substrates, or the corresponding UDP-Gal analogues are not recognized by transporters for Golgi import.

**FIGURE 1:**
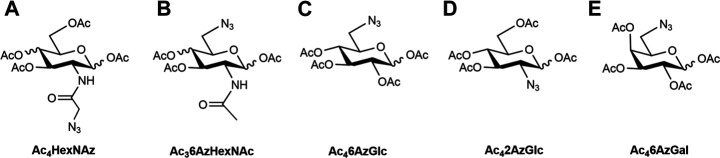
Structures of metabolic chemical reporters reported **(A**–**D)** and MCR **(E)** used in this study.

Here, we challenge the MCR per-O-acetylated-6-Azido-6-deoxy-Galactose (Ac_4_6AzGal, Figure 1) for metabolic labeling of intracellular O-glycosylation proteins since the success labeling of Ac_4_6AzGlc mediated with substrate promiscuity of OGT. We find that a range of mammalian cells are robustly labeled by treatment with Ac_4_6AzGal and with minimal toxicity to cells. Notably, *in vitro* experiments demonstrate that UDP-6AzGal is not a substrate for OGT, and we hypothesize that this labeling is a result of UDP-6AzGlc incorporation. The results of selective deglycosylation reactions of O- and N-linked glycans further confirm that Ac_4_6AzGal labeling is O-linked glycans. Moreover, this labeling is sensitive to the level of OGT but not OGA, and knockdown of the enzymes of GALT or GALE remarkably reduce the labeling efficiency. Taken together, these results further prove that various protein incorporations by metabolic reporters are the results of interconvertion and promiscuity labeling.

## Results and Discussion

**FIGURE 2:**
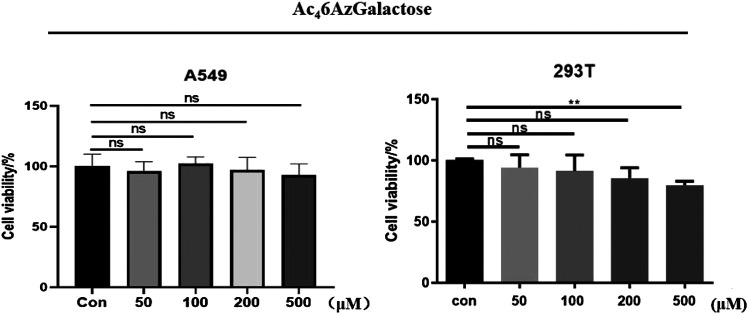
Viability of A549 and 293T cells was measured after treatment with various concentrations of Ac_4_6AzGal for 72 h using a CCK-8 cell-proliferation assay.

We synthesized Ac_4_6AzGal by the method previously reported (Chen and Withers, 2018). With the MCR in hand, the cytotoxicity was first evaluated using CCK8 cell proliferation experiments. We find that there is no effective cell loss even at a concentration of 500 μM after treatment with different concentrations of Ac_4_6AzGal to HEK293 cells and A549 cells for 72 h (Figure 2). Moreover, there is also no influence on the A549 cellular morphology treatment with the highest concentration tested (Supplementary Figure S1). To our best of knowledge, galactose is a ubiquitous component of cellular surface glycans including N- and O-glycans and several forms of glycolipids, and these glycans are always connected with biological activities. Any influence on the cell biological functions after incubation with unnatural galactose are reported because some monosaccharide analogues are potential inhibitors in an unexpected way (Macauley et al., 2014; Geissner et al., 2021). The scratch test results demonstrated that no obvious effect on the migration is observed in A549 cell after treatment with a range of concentrations of Ac_4_6AzGal for 36 h (Supplementary Figure S2). Together, these results suggest that Ac_4_6AzGal is safe for further investigation.

**FIGURE 3:**
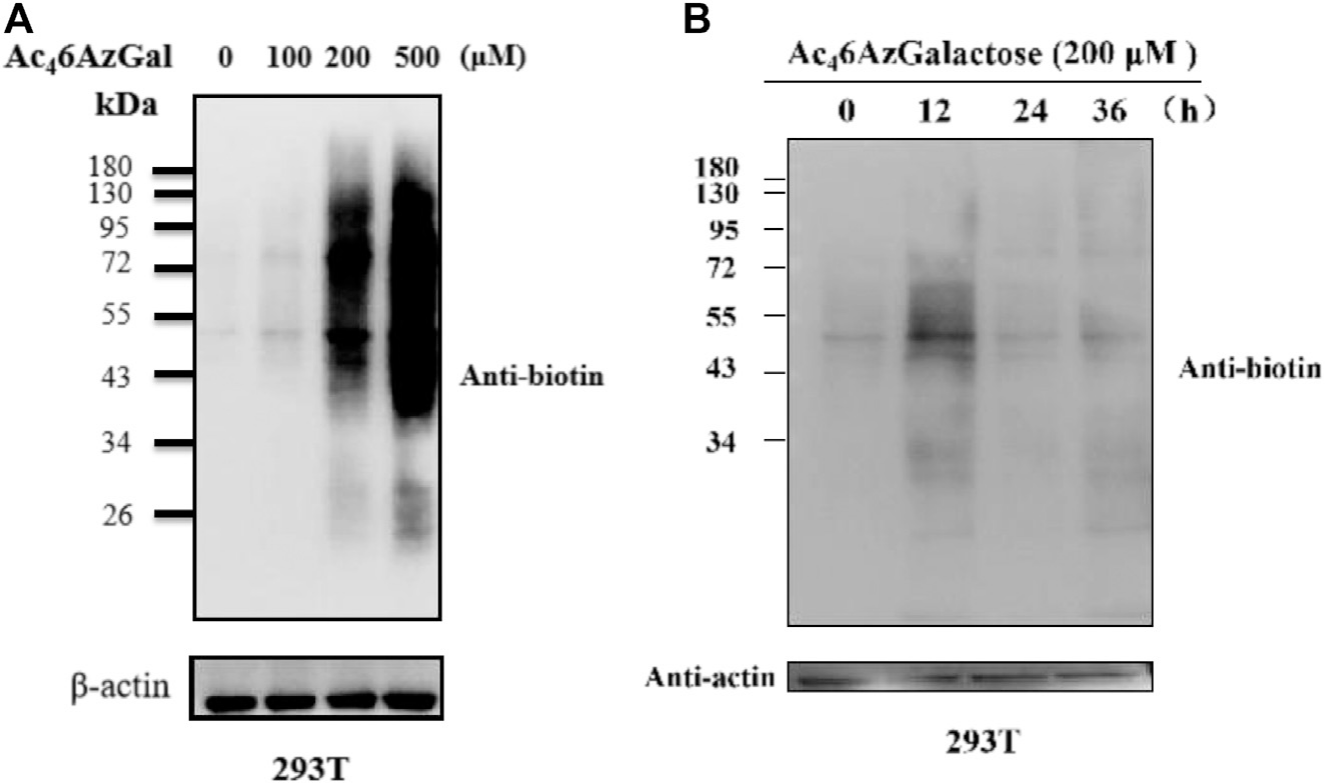
293T cells were treated with varying concentrations of Ac_4_6AzGal for 16 h **(A)** or with 200 μM Ac_4_6AzGal for the indicated times **(B)**, followed by CuAAC and analysis by Western blot.

Next, to further characterize Ac_4_6AzGal, 293T cells were treated with various concentrations of Ac_4_6AzGal for 16 h, which were then lysed and normalized using a Protein quantification kit. Equal amounts of proteins were subjected to a copper-catalyzed azide–alkyne cycloaddition reaction (CuAAC) and analyzed by Western blot using streptavidin-HRP for biotin labeling. The results of Western blot indicate that dose dependence of labeling is observed with a similar pattern and remarkable labeling at the concentration of 500 μM (Figure 3A). As far as we know, metabolic reporters only indicate modifications that occur in the labeling time, raising the attention when labeling proteins were needed for isolation and characterization. Subsequently, to determine the kinetics of protein labeling, 293T cells were treated with Ac_4_6AzGal (200 μM) for different lengths of time; then, performed cells were lysed and reacted with the Biotin-PEG_4_-Alkyne tag, and Western blot analysis was performed (Figure 3B). The Ac_4_6AzGal-dependent labeling is highly detectable at 12 h with gradual degradation during the next 24 h, raising the possibility that detection or isolation of Ac_4_6AzGal-labeled proteins should be harvested within an appropriate time. Together, the results above demonstrate that Ac_4_6AzGal is an effective intracellular protein MCR in a dose- and time-dependent manner.

**FIGURE 4:**
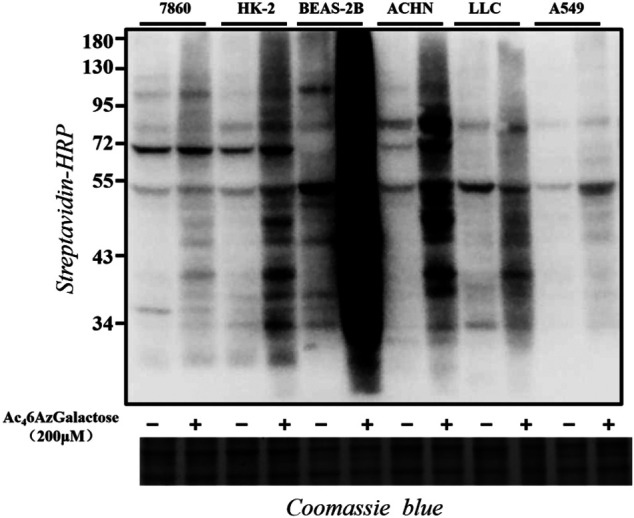
Proteins in different cell lines are labeled upon Ac_4_6AzGal treatment.

In order to explore the generality of Ac_4_6AzGal as a metabolic reporter, we tested a panel of different mammalian cell lines. Specifically, 7860, HK-2, BEAS-2B, ACHN, LLC, and A549 cells were selected for treatment with Ac_4_6AzGal (200 μM) for 16 h. The cells were then lysed and reacted with CuAAC. Western blot analysis results show that Ac_4_6AzGal enables efficient labels in different cell lines with a diversity pattern and intensity of modified proteins (Figure 4). The different labeling profiles between BEAS-2B and A549 cells further reveal the marking selectivity of the metabolic chemical reporter in various cell lines.

**FIGURE 5:**
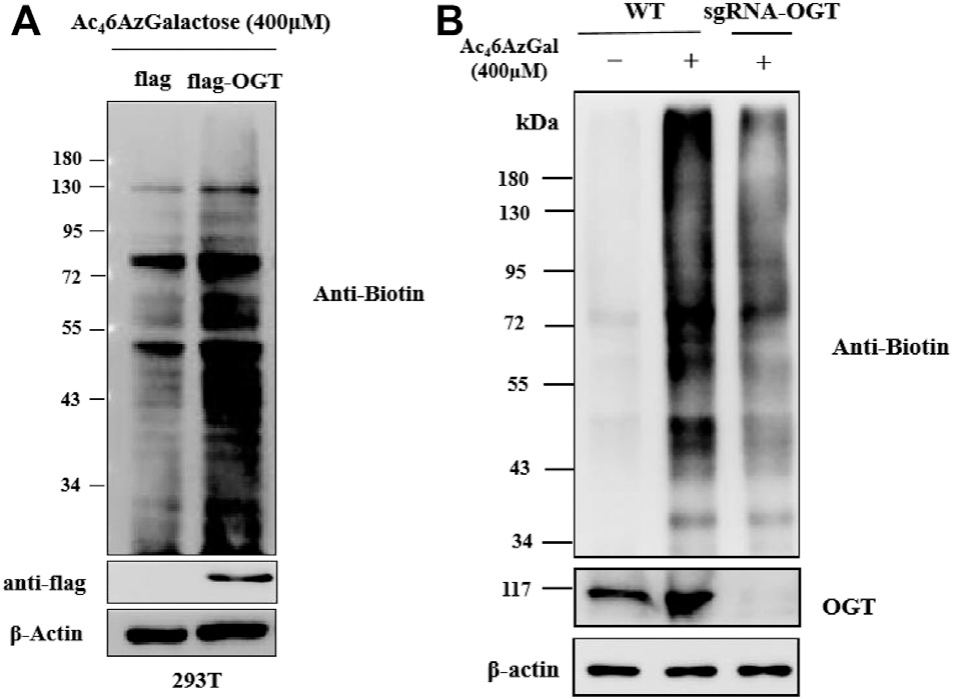
Protein labeling by Ac_4_6AzGal is dependent on the expression of OGT. **(A)** Over-expressed OGT could enhance the labeling efficiency of Ac_4_6AzGal and **(B)** Knock out OGT would decrease the labeling in 293T cells.

Ac_4_6AzGlc has been reported as a substrate of OGT and could be used for robust labeling of H1299 lysates in the presence of OGT and UDP-6AzGlc; however, the labeled profile is irrelevant with the enzyme of glutamine fructose-6-phosphate aminotransferase (GFT) in the hexosamine biosynthetic pathway (HBP) and OGA (Darabedian et al., 2018). We are interested in examining the labeling performances of Ac_4_6AzGal, which are related to the activity of OGT and OGA. Therefore, we first detected if the UDP-6AzGal donor is a substrate of OGT. A one-pot reaction containing UDP-6AzGal, the YAVVPVSK peptide, and OGT was performed as the method we previously reported (Li et al., 2018). Unfortunately, no 6AzGal modified peptide was detected compared to the UDP-GlcNAc group, which is a natural substrate for OGT and almost complete transformation to the GlcNAc-peptide (Supplementary Figure S3). In light of the incorporation results given above by Ac_4_6AzGal, we hypothesized that this labeling may be the result of UDP-6AzGlc through the interconversion of UDP-6AzGal by GALE in the Leloir pathway since UDP-6AzGlc has been reported as a substrate of and regulated by OGT (Darabedian et al., 2018). Therefore, we subsequently performed transient transfection of HEK293 cells with Flag-expressed OGT and Flag-labeled empty plasmids for 48 h. These cells were then treated with Ac_4_6AzGal for 16 h, followed with lysed with biotinylated proteins *via* a click reaction and analyzed by Western blot. We find that a higher labeling band is obtained in OGT-overexpressing cells, which shows that OGT can transfer Ac_4_6AzGal to mammalian proteins after metabolic biosynthesis (Figure 5A). Meanwhile, in order to further confirm the role of OGT in the labeling of Ac_4_6AzGal, the OGT knockdown cell line of B16 previously constructed by our group was incubated with Ac_4_6AzGal for 16 h and normal B16 cells were used as the control. After harvesting, the lysates were subjected to CuAAC reaction, and the Western blot results demonstrate decreased labeling in the OGT knockdown cell line, indicating that low expression of OGT reduces the modification of proteins by 6AzGal (Figure 5B).

**FIGURE 6:**
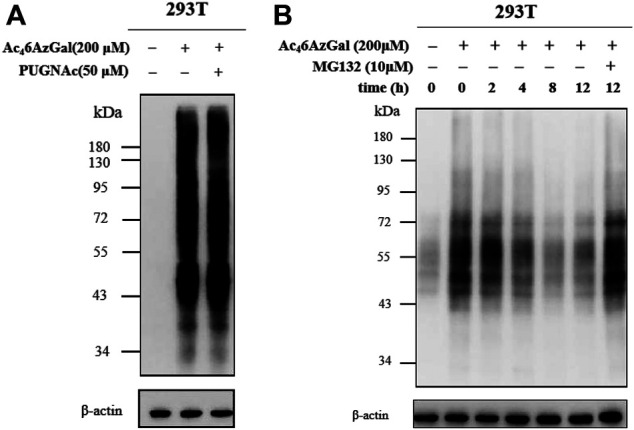
Ac_4_6AzGal labeling is not mediated by OGA. **(A)** OGA inhibitor has no effect in the protein labeling of Ac_4_6AzGal; **(B)** Pulse-chase experiment indicates Ac_4_6AzGal labeling is degraded with the dependence of time.

Based on our description of the O-GlcNAcylated process in the preface, we then explored whether Ac_4_6AzGal was also a kind of reversible modification. HEK293 cells were treated with PUGNAc (50 μM, OGA inhibitor) in the presence of Ac_4_6AzGal for 16 h and then treated CuAAc with azide biotin-alkyne, and Western blot analysis was performed (Figure 6A). Analysis results show no significant enhancement of 6AzGal labeling, showing that OGA is not responsible for the removal of the modification from proteins. To the best of our knowledge, metabolic reporters of O-GlcNAc modification have the ability to read out on the turnover of protein modifications using a pulse-chase format. Therefore, to further prove that Ac_4_6AzGal is dynamically incorporated into O-linked proteins, 293T cells were first treated with 200 μM Ac_4_6AzGal for 16 h. The cells were washed and changed with fresh media, which were collected after different lengths of time, lysed, and subjected to CuAAC with Biotin-PEG_4_-Alkyne. Western blot analysis shows a steady loss of protein labeling over the course of time, indicating that Ac_4_6AzGal causes O-linked modifications and can be degraded by the proteasome since OGA is not responsible for the removal (Figure 6B). To further determine if the reduction in labeling was due to protein turnover, we repeated this pulse-chase experiment in the presence of the proteasome inhibitor MG132 (10 μM). Analysis by Western blot shows some stabilization in the signal upon MG132 treatment, demonstrating that the modified proteins are resulted from degradation. Together, these results show that Ac_4_6AzGal labeling is, at least partially, conducted by OGT but not OGA, which further supports the possibility that Ac_4_6AzGal is a potential substrate in the process of intracelluar modification.

**FIGURE 7:**
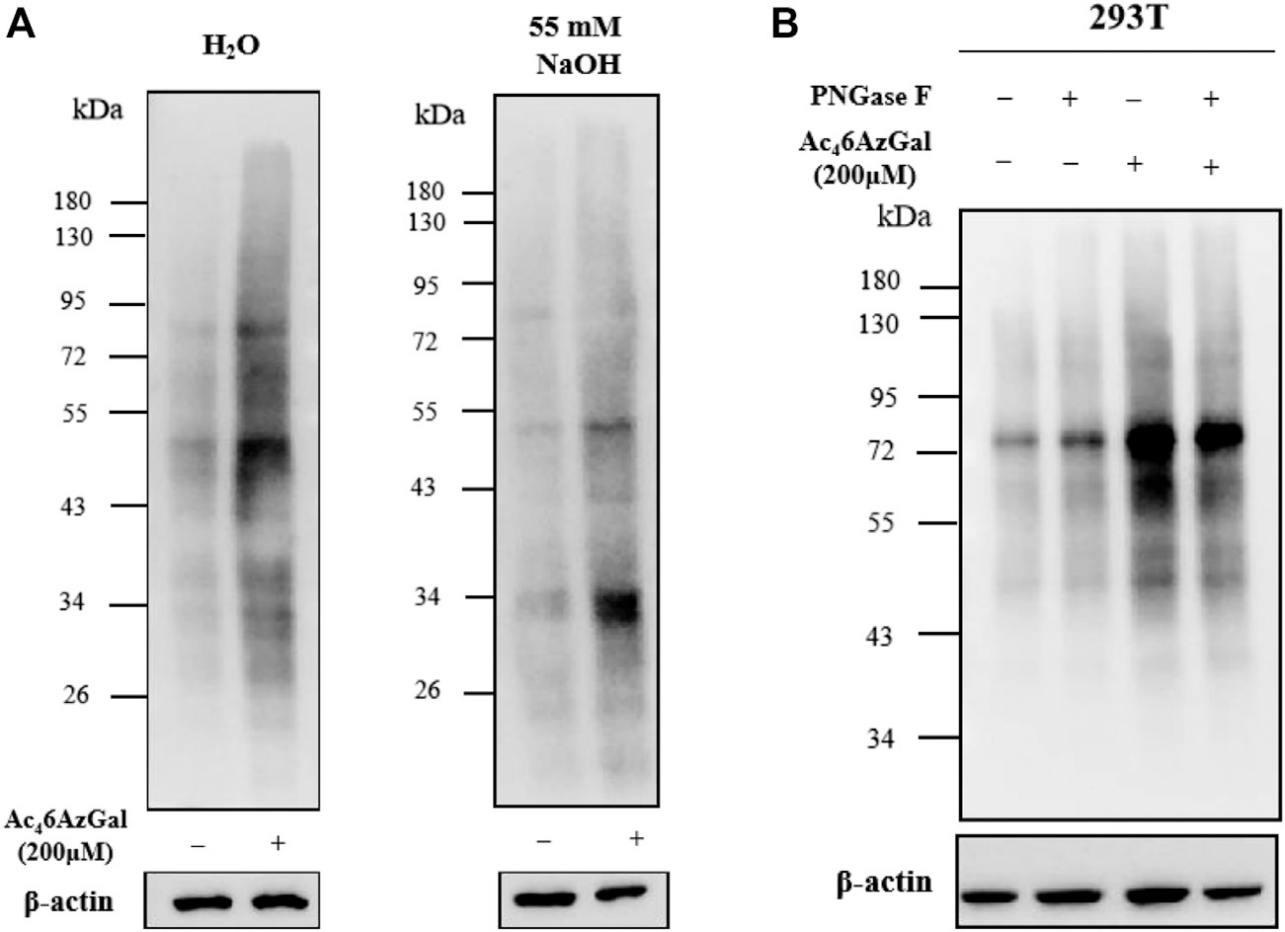
Ac_4_6AzGal labels intracellular O-linked proteins rather than N-linked surface glycans. **(A)** Ac_4_6AzGal is not incorporated into the N-linked glycans; **(B)** Ac_4_6AzGal signal is derived from O-linked glycoproteins.

In order to confirm whether the intracellular labeling by Ac_4_6AzGal is O-linked, *β*-elimination and enzymatic cleavage of N-linked glycans using PNGase-F were employed. 293T cell lysates were obtained after treatment with 200 μM Ac_4_6AzGal for 12 h, followed by CuAAC with Biotin-PEG_4_-Alkyne, separation by sodium dodecyl sulfate polyacrylamide gel electrophoresis, and transfer to PVDF membranes, and duplicate samples were prepared. One membrane was treated with H_2_O as the control, and the other one was incubated with 55 mM NaOH to induce *β*-elimination and kept at 40°C for 24 h. A notable fraction of the 6AzGal-dependent signal is lost, which suggested that this incorporation is O-linked and sensitive to *β*-elimination (Figure 7A). Similarly, 293T cells were treated with either 200 μM Ac_4_6AzGal or the dimethyl sulfoxide (DMSO) vehicle for 12 h. The corresponding cell lysates were then incubated with H_2_O or PNGase-F at 37°C for 6 h, followed by CuAAC with Biotin-PEG_4_-Alkyne and Western blot analysis. The results demonstrate that no significant loss in the signal is observed, suggesting that Ac_4_6AzGal is not incorporated into N-glycans (Figure 7B). Together, these discoveries confidently indicate that Ac_4_6AzGal protein labeling is in the O-linked form, which perfectly fills the gaps that galactose could label surface glycans by microinjecting with UDP-6AlGal, and in the case of intracellular proteins, labeling could be achieved using MCR Ac_4_6AzGal after metabolic labeling.

**FIGURE 8:**
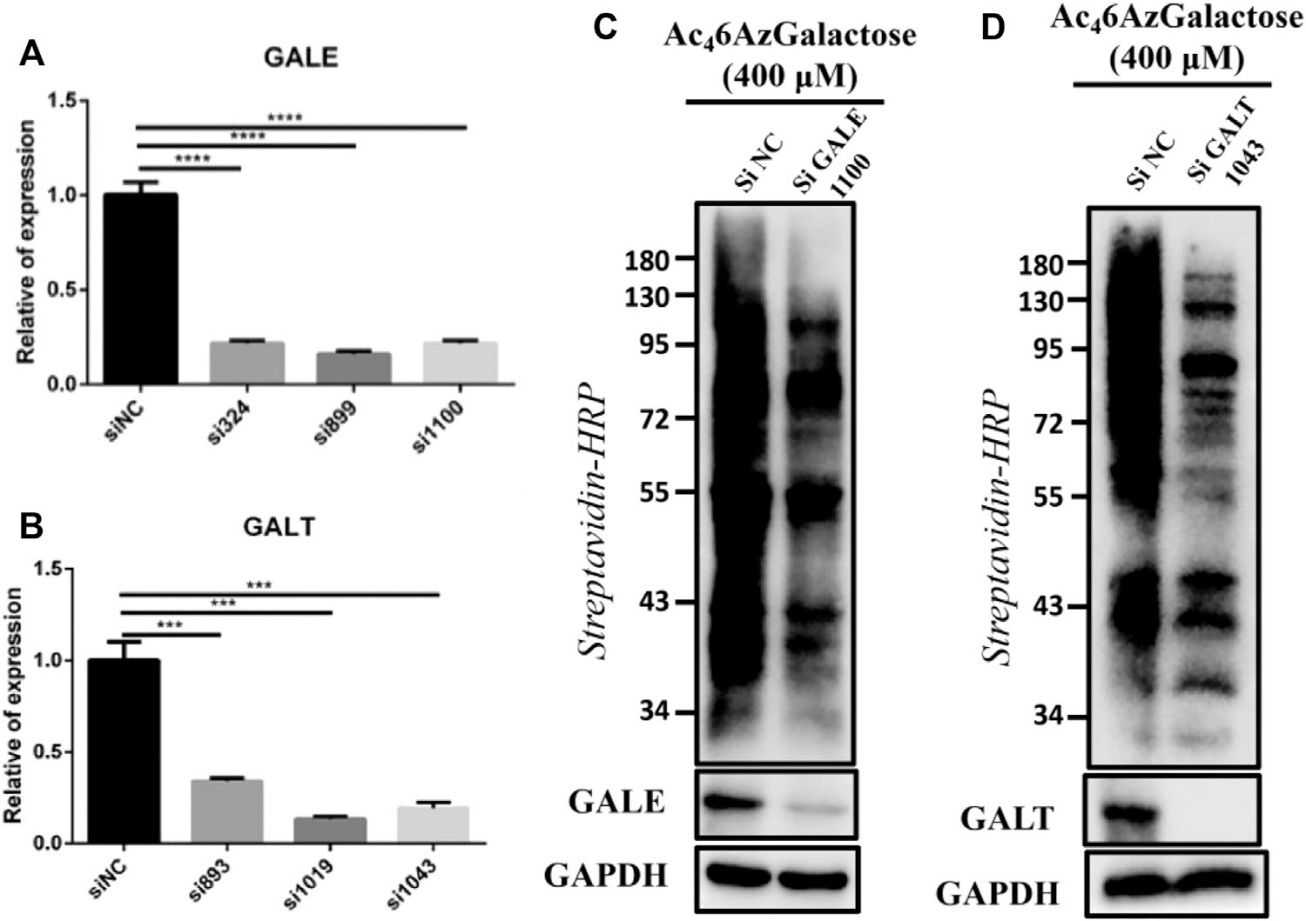
Characterizing the roles of GALT and GALE in the labeling of Ac_4_6AzGal. Knocking down the enzyme expressions of GALE **(A)** and GALT **(B)** by siRNA; The influences on Ac_4_6AzGal labeling after inferring the expression of GALE **(C)** and GALT **(D)**.

Finally, we examined the roles of key enzymes, GALT and GALE, in the production of UDP-6AzGal via the galactose metabolism pathway. The RNA expressions of interested enzymes were interfered by adding the corresponding siRNA. After successfully interfering the expressions of GALT and GALE (Figures 8A,B), one siRNA was selected from each group for further experiments, termed siGALE 1100 and siGALT 1043, respectively, with siNC as the control. HEK293 cells were previously treated with siRNA for 48 h and then subjected to incubation with Ac_4_6AzGal for another 16 h, followed by lysis and CuAAC with Biotin-PEG_4_-Alkyne. Notably, in comparison to the control, a robust loss of the labeling signal in each group demonstrates that both the enzymes of GALT and GALE play essential roles for Ac_4_6AzGal in the metabolic labeling in cells (Figures 8C,D). It is noted that there is an incomplete loss of labeling when knocking down GALT and GALE. We reason that this possibility of residue protein labeling, on one hand, is the products that are not reversed by OGA; on the other hand, it is likely that Ac_4_6AzGal occurs with unexpected S-glyco-modification, an atypical glycosylation, between protein cysteines and per-O-acetylated sugars, which has attracted great attention for the metabolic glycan labeling (Qin et al., 2018; Hao et al., 2019; Qin et al., 2020). Of course, glycation, which is characterized by the condensation of the aldehyde form of monosaccharides with nucleophilic amino acid side chains, may have also occurred. We next evaluated the possible induction of S-glycosylation by a range of concentrations of Ac_4_6AzGal according to the method reported (Hao et al., 2019). Briefly, chemical reporters including Ac_4_GlcNAz, Ac_4_GalNAz, and Ac_4_6AzGal were incubated with the B16 cell lysate at 37°C for 2 h. After labeling through click chemistry, Western blot analysis indicates that both Ac_4_GlcNAz and Ac_4_GalNAz afford robust labeling intensity in accord with the results discovered by Chen’s group (Supplementary Figure S4). Moreover, Ac_4_6AzGal exhibits slight labeling intensity, to some extent, suggesting that nonspecific S-glyco-modification happens.

In conclusion, galactose is a naturally occurring monosaccharide used to build complex N-/O-glycans on the surface of vertebrates and is metabolized predominantly *via* the Leloir pathway. The imparied function of the Leloir pathway of the galactose metabolism results in galactosemias. Metabolic chemical reporters of glycosylation in combination with bio-orthogonal reactions are widely used for the identification and visualization of glycoconjugates. However, as far as we know, there is no unnatural galactose, such as Ac_4_2AzGal, Ac_4_4AzGal, and Ac_4_6AzGal, which could be transferred to the corresponding uridine diphosphate galactose analogue for further metabolic labeling of cell-surface glycans due to the intolerance of unnatural galactose in the related synthetic pathway. Here, we turn our attention to the intracellular labeling by using per-O-acetylated-6-Azido-6-deoxy-Galactose (Ac_4_6AzGal) to provide further insight into the tolerance of biosynthetic enzymes and glycosyltransferases to Ac_4_6AzGal. We demonstrate that Ac_4_6AzGal is able to label intracellular proteins by treatment with a variety of mammalian cells. Notably, the pattern of this labeling is conducted by OGT but is not related to OGA, which further reveals the substrate promiscuity of OGT. However, substrate specificity of OGT is detected for UDP-6AzGal, and the results demonstrate that UDP-6AzGal cannot be recognized by OGT. Therefore, we infer that the protein labeling of 6AzGal intracellular is incorporated in the form of UDP-6AzGlc instead of UDP-6AzGal, and the two donors are interchangeable *via* the enzyme in the Leloir pathway. Moreover, the enzymes of galactose-1-phosphate uridylyltransferase (GALT) and UDP-galactose 4′-epimerase (GALE) are also confirmed to regulate the efficiency labeling of Ac_4_6AzGal, suggesting that the enzymes in the Leloir pathway, at least partially, participate in the metabolic biosynthesis of Ac_4_6AzGal. These results support the conclusion that galactose analogues can modify endocellular proteins, and is significant on intracellular protein labeling under certain conditions that involve deficiency of the galactose metabolism.

## Data Availability

The original contributions presented in the study are included in the article/Supplementary Material, and further inquiries can be directed to the corresponding authors.
