# Supplementary material for: The Metabolic Chemical Reporter Ac46AzGal Could Incorporate Intracellular Protein Modification in the Form of UDP-6AzGlc Mediated by OGT and Enzymes in the Leloir Pathway
Source: Front Chem. 2021 Oct 12;9:708306. doi: 10.3389/fchem.2021.708306 (PMC8546251; doi:10.3389/fchem.2021.708306)
Supplement: Supplementary file 1 [file DataSheet1.PDF]

Supplementary material

The Metabolic Chemical Reporter $Ac_46AzGal$ Could Incorporate Intracellular Protein Modification in the form of UDP-6AzGlc that Mediated by OGT and Enzymes in Leloir Pathway

Jiajia Wang [a,b,#], Biao Dou [a,#], Lu Zheng [a,#], Wei Cao [a], Peiyu Dong [a], Yingyi Chen [a], Xueke Zeng [a], Yinhang Wen [a], Wenxuan Pan [c], Jing Ma [c,*], Jingying Chen [a,*], Xia Li [a,*]

[a] *Joint National Laboratory for Antibody Drug Engineering, The first affiliated hospital of Henan University, School of Basic Medicine Science, Henan University, 475004, Kaifeng, China*

[b] *State Key Laboratory of Medicinal Chemical Biology, Nankai University, Haihe Education Park, 38 Tongyan Road, Tianjin, 300353, China*

[c] *School of Pharmacy, Institute for Innovative Drug Design and Evaluation, Henan University, 475004, Kaifeng, China*

Cell Culture.

HEK239T and A549 cells were cultured in DMEM media(Sigma) supplemented with 10% Fetal Bovine Serum (PAN Seratech), 100 U/mL penicillin and 100 mg/mL streptomycin 7860, BEAS-2B and LLC cells were cultured in RPMI media (Sigma) supplemented with 10% Fetal Bovine Serum (PAN Seratech), 100 U /mL penicillin and 100 mg/mL streptomycin. ACHN and HK-2 cells were cultured in MEM media (Procell) supplemented with 10% Fetal Bovine Serum (PAN Seratech), 100 U/mL penicillin and 100 mg/mL streptomycin. All cell lines were maintained in a humidified incubator at 37 °C and 5.0% CO_2.

CCK-8.

HEK293 cells and VERO1 cells were seeded at a density of 1000 cells per well into 96-well plates for 4 h, and then treated with 0, 50, 100, 200， 500 and 1000 μM $Ac_46AzGalactose$ for 48h under low glucose medium conditions (1 g/L). Then 10 μL of Cell Counting Kit-8 (CCK-8) solution was added to each well and incubated for 2h at 37 °C. Then monitored using a microplate reader at a wavelength of 450 nm.

Metabolic Labeling.

To cells at 75−80% confluency, media was exchanged for fresh media containing

$Ac_46AzGalactose$ (200 μM) (1000× stock in DMSO), or DMSO vehicle was added as indicated. Preparation of soluble protein cleavage products. The cells were collected by trypsinization and pelleted by centrifugation at for 5 min at 3000g, followed by washing 1× with PBS (1 mL). Cell pellets were then resuspended in 200 μL of 1% NP-40 lysis buffer [1% NP-40, 150 mM NaCl, 2 mM $MgCl_2$, 10% glycerol,TritonX-100 and 50mM Tris pH 7.4] containing 1% protease inhibitor and 1% PMSF for 30 min and then centrifuged for 20 min at 12 000rpm at 4°C. The supernatant (soluble cell lysate) was collected and the protein concentration was determined by BCA assay (Pierce, ThermoScientific).

Cu(I)-Catalyzed [3+2] Azide−Alkyne Cycloaddition (CuAAC). 200 μg cell lysate was diluted with lysis buffer to a final concentration of 1 μg μL^{-1}. The newly prepared click chemistry cocktail, containing 100 μM Biotin-PEG_4-Alkyne, 1 mM sodium ascorbate, 100 μM Tris 3-hydroxypropyltriazolylmethyl) amine and 1 mM $CuSO_4{\cdot}5H_2O$. The reaction was gently vortexed and allowed to sit at room temperature for 2 h. After the reaction, 5 times the volume of ice methanol was added and the protein was precipitated overnight at - 80 ℃. The reaction mixture was then centrifuged at 10000g for 10 min at 4 °C. Wash with ice methanol twice, discard the supernatant, and air-dry the protein sample for 30 minutes and dissolved in 40 μL of 4% SDS buffer (4%SDS, 150 mM NaCl, 50 mM TEA, pH 7.4). The mixture was sonicated in a bath sonicator to ensure complete dissolution,and 10 μL of 5 × SDS free loading buffer (50% glycerol, 0.5% bromophenol blue, and 3.5% β-mercaptoethanol, pH 6.8) was then added. Thesample was boiled for 10 min at 100°C, and 20 μg of protein was loaded per lane for SDS-PAGE separation.

Enzymatic synthesis of glycopeptide with OGT and UDP-6AzGal

To determie the substrate specificity of UDP-6AzGal to OGT, we followed the procedure reported by our group. Briefly, a total volume of 200 μL solutions containing 2 mM peptide, 6 mM UDP-GlcNAc analogs, 100 μg ncOGT in buffer (50 mM Tris-HCl, pH 7.5, 10 mM Mg^{2+}) were incubated at 37°C for 2 h. The reaction mixtures were boiled for 10 min and centrifuged at 12, 000 g for 30 min to remove the enzyme. The reaction mixture (100 μL) was diluted with 100 μl de-ionized water and ready for HPLC separation.

Deglycosylation of *N*-glycans with PNGase F.

A total volume of 200 μL buffer containing 200 μg 293T cells lysates, 6 μL of PNGase F or 6μL of water, the mixture was incubated for 6 h at 37 °C. Then the reaction was added 250 μL of water, 400 μL of methanol and 150 μL of chloroform to remove the enzyme by protein precipitation. Samples were vortexed and centrifuged at 16,000 x g at 4 °C for 5 min. The aqueous layer was removed, and 1 mL of ice-cold methanol was added. Samples were centrifuged at 16,000 g at 4 °C for 3 min and supernatant was discarded and the precipitation was washed twice by 500 μL of ice-cold methanol and centrifuged at 16,000 g at 4 °C for 3 min. Methanol was discarded and protein pellet was dried at 37 °C for 5 min.

Samples were diluted with lysis buffer (1% NP-40, 150 mM NaCl, 50 mM triethanolamine, pH 7.4) to obtain a desired concentration of 1μg/μL. The mixture was added 2 μL with 50 mM CuSO4, 6 μL 10 mM THPTA (in water), 2 μL 100 mM biotin-PEG4-Alkyne (in DMSO), and 10 μL 50 mM sodium ascorbate (in water, made fresh) for a total reaction volume of 200 μL. The reaction was genty vortexed for 2 h at room temperature. Samples were then precipitated as before and solubilized in 80 μL 4% SDS buffer (4% SDS, 150 mM NaCl, 50 mM TEA, pH 7.4) and 20

μL 5X SDS loading buffer. Samples were analyzed by SDS-PAGE and Western blot with streptavidin-HRP and detection with Clarity ECL solution.

***β*-elimination of O-glycans metabolically labeled by $Ac_46AzGal$.**

293T cell lysates (200 μg) were diluted with lysis buffer (1% NP-40, 150 mM NaCl, 50 mM triethanolamine TEA, pH 7.4) to obtain a desired concentration of 1μg/μL. The mixture was added 2 μL with 50 mM CuSO4, 6 μL 10 mM THPTA (in water), 2 μL 100 mM biotin-PEG4-Alkyne (in DMSO), and 10 μL 50 mM sodium ascorbate (in water, made fresh) for a total reaction volume of 200 μL. The reaction was gently vortexed for 2 h at room temperature. The reaction was added 250 μL of water, 400 μL of methanol and 150 μL of chloroform to precipitate protein. Samples were vortexed and centrifuged at 16,000 x g at 4 °C for 5 min. The aqueous layer was removed, and 1 mL of ice-cold methanol was added. Samples were centrifuged at 16,000 g at 4 °C for 3 min and supernatant was discarded and the precipitation was washed twice by 500 μL of ice-cold methanol and centrifuged at 16,000 g at 4 °C for 3 min. Methanol was discarded and protein pellet was dried at 37 °C for 5 min. Samples were then dissolved in 80 μL 4% SDS buffer (4% SDS, 150 mM NaCl, 50 mM TEA, pH 7.4) and 20 μL 5X SDS loading buffer. Samples were analyzed by SDS-PAGE and transferred onto PVDF membranes. One replicate membrane was placed in a sealed bag with 55 mM NaOH and the other membrane was placed in a sealed bag with water. The membranes were incubated at 40 °C for 24 h. The membranes were rinsed with water to remove any residual sodium hydroxide followed by incubation with TBS-T for 5 min. The membranes were blocked with 5% w/v BSA in TBS-T for 1 h at room temperature then incubated with 1:25,000 dilution of streptavidin-HRP in TBS-T with 5% w/v BSA for 1 h. Solution was removed, and the membranes were rinsed three times with TBS-T for 7 min followed by one rinse with TBS for 5 min. The membranes were incubated with Clarity ECL solution for 5 min, and then the chemiluminescence was imaged.

RNA interference.

293T cells with ~60% confluence in six-well plates were transfected with 50 nM SiNC, SiGALT and SiGALE(Gene Pharma) using the RNA Mix transfection reagent (Invitrogen, CA, USA) according to the manufacturer's instructions, and the siRNA sequences were as follows:
5′-GGAAGAAGAUCCCGUACAATT-3′(sense) and
5′-UUGUACGGGAUCUUCUUCCTT-3′(antisense) for siGALE;
5′-GCUACGAAAUGCUUGCUCATT-3′(sense) and
5′-UGAGCAAGCAUUUCGUAGCTT-3′(antisense) for SiGALT; and
5′-UUCUCCGAACGUGUCACGUTT-3′(sense) and
5′-ACGUGACACGUUCGGAGAATT-3′(antisense) for SiNC.After 48h,knock down of GALT and GALE were confirmed via western blot.

Western Blotting.

Proteins were separated by SDS-PAGE before being transferred to NC membrane using standard Western blotting procedures.All Western blots were blocked in TBST (0.1% Tween-20, 150 mM NaCl, 10 mM Tris pH 8.0) containing 5% nonfat milk for 2 h at rt. The blots were then incubated with an appropriate primary antibody in blocking buffer for 2 h at rt. DM-17 was used at 1:1000 for detection of OGT. The streptavidin-HRP antibody was used at 1:10000. The blots

were then washed three times in TBST for 10 min and incubated with the horseradish peroxidase (HRP)-conjugated secondary antibody for 2h in blocking buffer at RT. HRP conjugated anti-mouse secondary antibody was used at 1:10000 dilution. After being washed three more times with TBST for 10min, the blots were developed using ECL reagents.

Migration Assay.

The effect of $Ac_4$6AzGalactose on the lateral migration of 293T cells was analyzed with scratch test. For cell scratch test, HEK293T were seeded on 6-well plates and grown up to 90% confluence in complete DMEM medium. A proportion of cells was removed from the mono layer with a sterile pipette tip (about 0.5 mm in width) and replaced with DMEM medium containing 200 μM $Ac_4$6AzGalactose, and the scratch images were captured at the beginning and at a variety of time points. Images were quantified to compare the migration rate of different cell groups with IPP6.0 image analysis software at 200×field.

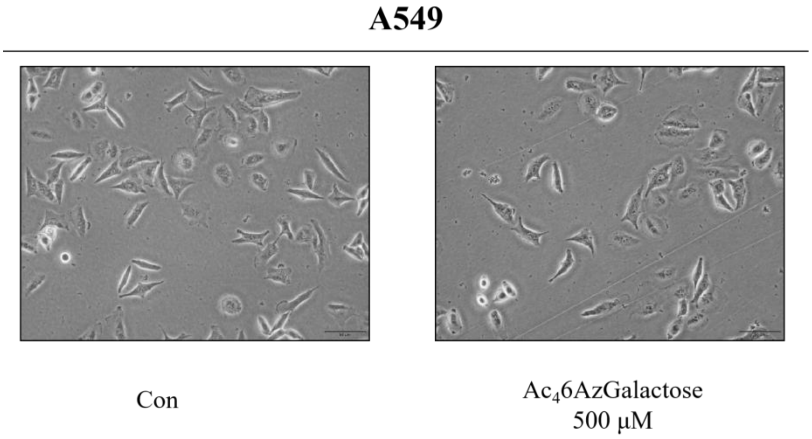

Figure S1. Comparison of A549 cellular morphology after treatment with or without 500 μM $Ac_4$6AzGal.

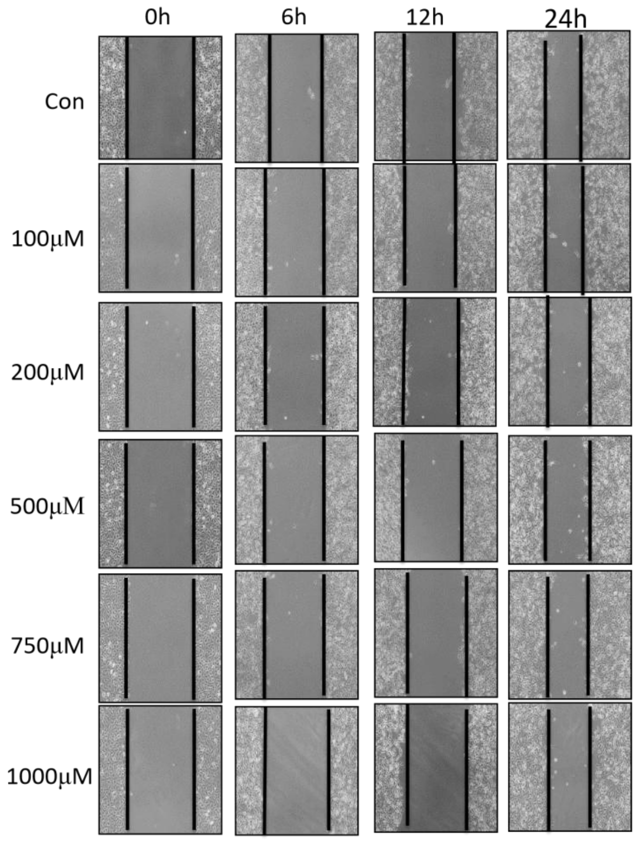

Figure S2. The scratch tests with a range of concentration of Ac46AzGal for 36 h.

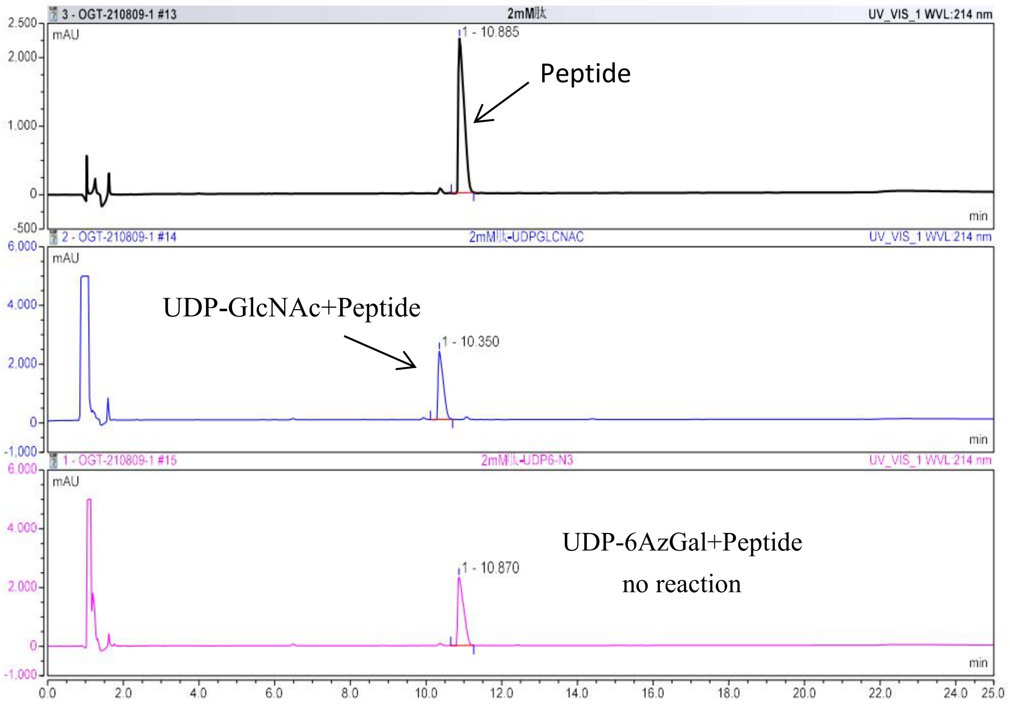

Figure S3. The HPLC profile for OGT reactions toward UDP-GlcNAc and UDP-6AzGal substrates.

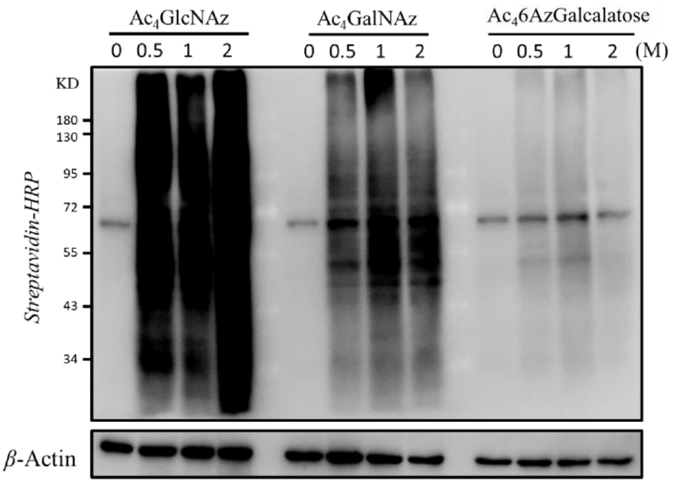

Figure S4. Comparison of artificial cysteine S-glycosylation induced by per-O-acetylated unnatural monosaccharides with a range of concentration of Ac_4GlcNAz, Ac_4GalNAz and $Ac_4$6AzGal at 0 M, 0.5 M, 1 M or 2 M, respectively, for 36 h.
